# Determinants of HIV, viral hepatitis and STI prevention needs among African migrants in Germany; a cross-sectional survey on knowledge, attitudes, behaviors and practices

**DOI:** 10.1186/s12889-015-2098-2

**Published:** 2015-08-06

**Authors:** Claudia Santos-Hövener, Ulrich Marcus, Carmen Koschollek, Hapsatou Oudini, Mara Wiebe, Omer Idrissa Ouedraogo, Adama Thorlie, Viviane Bremer, Osamah Hamouda, Marie-Luise Dierks, Matthias an der Heiden, Gérard Krause

**Affiliations:** Department for Infectious Disease Epidemiology, Division for HIV/AIDS, STI and Blood-borne Infections, Robert Koch Institute, Seestr. 10, 13353 Berlin, Germany; AIDS Foundation Hamburg, Lange Reihe 32, 20099 Hamburg, Germany; Youth Migration Service Hamburg, AWO Schleswig-Holstein, 20099 Hamburg, Germany; Institute for Epidemiology, Social Medicine and Health Systems Research, Hanover Medical School, Carl-Neuberg-Straße 1, 30625 Hanover, Germany; Department of Epidemiology, Helmholtz Centre of Infection Research, Inhoffenstr. 7, 38124 Braunschweig, Germany; Hannover Medical School, Hannover, Germany

**Keywords:** Migrants from sub-Saharan Africa, KABP, Community-based participatory research, HIV, Viral hepatitis, STI

## Abstract

**Background:**

Migrants from sub-Saharan Africa (MisSA) are a relevant sub-group for HIV-transmission in Germany. A total of 10-15 % of all newly diagnosed cases are MisSA, and approximately one third acquired HIV in Germany. There is limited information on knowledge, attitudes, behaviors and practices (KABP) regarding sexual health in African communities residing in Germany.

**Methods:**

From October-December 2013 we conducted a cross-sectional survey on KABP regarding HIV, viral hepatitis (HEP), and sexually transmitted infections (STI) among MisSA in Hamburg as a community-based participatory research project to identify knowledge gaps, sexual risk behavior regarding HIV/HEP/STI, HIV/STI-testing history and attitudes toward people living with HIV (PLWH). Trained peer researchers recruited participants through outreach. Questionnaires in German, English or French were either administered face-to-face or self-completed. Questions on knowledge about HIV/HEP/STI presented true statements; participants were asked if they knew the information before. To detect differences in sub-groups, unadjusted odds ratios (OR) were calculated, and a multivariate analysis for knowledge on HIV/HEP/STI was performed.

**Results:**

The final sample included 569 participants of whom 57 % were men. Most participants originated from Western and Central sub-Saharan Africa. Median time living in Germany was 6 years. Overall, 28 % had a university degree and 54 % reported a good level of German language. Over 80 % knew the risks for HIV transmission. A total of 44 % of respondents wrongly assumed that an HIV-diagnosis might lead to deportation and 64 % were not aware of the free and anonymous local HIV/STI-testing service. The proportion of participants with knowledge of presented facts on HEP varied from 40-58 %. The respective proportion on STI was 28-68 % and better among women compared to men (44 % vs. 54 %; OR = 1.45; 95 % CI 1.22-1.74). Men reported more often casual sex partners than women (43 % vs. 23 %; OR = 2.6; 95 % CI 1.7-4.0), and more frequently a previous STI (58 % vs. 39 %; OR = 2.1; 95 % CI 1.1-4.1). Overall, 16 % of women reported a history of sexual violence. The majority of respondents (75 %) reported that they would treat PLWH like any other person.

**Conclusion:**

Study participants demonstrated good knowledge on HIV-transmission but knowledge gaps regarding HIV/STI-testing services, HEP and STI. This calls for targeted interventions providing more information about these topics in African communities in Hamburg and possibly also elsewhere.

**Electronic supplementary material:**

The online version of this article (doi:10.1186/s12889-015-2098-2) contains supplementary material, which is available to authorized users.

## Background

### HIV, Hepatitis B and C in German-based migrants from sub-Saharan Africa

Epidemiological studies from different Western European countries show that migration has an impact on the epidemiology of HIV and viral hepatitis [1]. Twelve countries reported that two thirds of HIV diagnoses with heterosexual transmission were among people from high prevalence countries [2]. In 2011, eighteen countries provided data to the European Center for Disease Control and Prevention (ECDC) on whether a reported hepatitis B case was imported (not acquired in reporting country), which was true for 53 % of all reported cases [3]. This shows that migrant populations are disproportionately affected by HIV and viral hepatitis and underlines the importance of surveillance for these infections.

The HIV epidemic in Germany, like elsewhere in Western Europe, is predominantly driven by infections between men who have sex with men (MSM) [4, 5]. However, heterosexual transmission (HET) plays an important role as well and has been increasing in 2012 and 2013 [4]. Among all newly diagnosed HET cases, 70 % are people with countries of origin other than Germany; and between 40-50 % of HET annually are migrants from sub-Saharan Africa (MisSA) [2, 6]. Since 2013 there has been an increase in diagnosed HIV cases particularly in female MisSA [4].

In the following we use the term MisSA for all persons who were born in countries of sub-Saharan Africa (WHO-Region). Based on population statistics the number of MisSA residing in Germany is approximately 200,000, [7] but does not include persons with German citizenship or those without legal residence status.

At the beginning of the HIV epidemic in Germany it was assumed that MisSA generally “import” their HIV infection from country of origin. However, surveillance data has shown that the proportion of HIV infections acquired in Germany, and thus being reachable for primary prevention, ranged from 15-28 % in 2009 to 2014 [4, 6]. However the proportion is likely to be higher: A study from the UK estimated the proportion of MisSA who acquired infection within the UK to be three times higher than figures resulting from clinicians’ reports [8].

HIV among MisSA in Germany is often diagnosed at a late clinical stage of HIV infection [6, 9], potentially due to barriers to HIV-testing or health care in general [10]. This might indicate that the proportion of undiagnosed MisSA is higher than in other sub-populations affected by HIV [6].

There is currently no information on incidence or prevalence of hepatitis B (HBV), hepatitis C (HCV) in MisSA residing in Germany. The reported prevalence of chronic hepatitis B in Western sub-Saharan Africa (where most MisSA residing in Germany are originating from) is up to 14 % [11] compared to an estimated 0.3 % in the German general population. Estimates indicate that the prevalence of HCV infections in sub-Saharan countries is approximately 3 % [11, 12], compared with an estimated prevalence of 0.3 % in the German general population [13].

### Behavioral surveillance in migrant populations

Originating from a high prevalence country does not necessarily increase the individual risk for acquiring HIV, unless sex partners are chosen from a group with higher HIV prevalence. However, studies show that migrants have an increased vulnerability for HIV infection, because access to HIV prevention, testing and counseling is often limited due to legal, cultural, socio-economic or language barriers [3, 10, 14]. Also, the migration process itself can impact HIV risk and access to care, because migrants might experience (sexual) trauma, discrimination and marginalization as well as problems with legal status [1, 3, 15].

Therefore, ECDC and WHO recommend collecting information on behavioral indicators such as knowledge gaps and risk behavior for prevention planning [15, 16]. However, in Germany data on knowledge, attitude, behavior and practice (KABP) of MisSA is scarce and the population is not sufficiently reached with KABP-surveys addressing the German general population. The level of knowledge on HIV, HEP and STI, as well as potential risk behaviors of MisSA, have so far only been assessed in studies with very small sample sizes [17, 18]. In order to reach MisSA in Germany with HIV prevention, we need to determine knowledge and potential risk behaviors from a sufficiently large sample of MisSA.

To address and overcome potential barriers and cultural misunderstandings, it is crucial to involve the target population in the development of study design and recruitment. In other European countries, community-based participatory research approaches have shown to be suitable to reach African communities with surveys on HIV [19–22]. This included involving community partners in study design planning and recruitment (peer researchers) [17, 19, 23].

Considering this, we planned a KABP-survey in Hamburg, the city with the biggest MisSA community [7] in Germany.

Our overarching research interest was to determine the specific HIV, HEP and STI prevention needs in MisSA residing in Hamburg, Germany.

The specific objectives of this study were to identify i)knowledge gaps regarding HIV, ii) viral Hepatitis and iii) STI; to describe iv) HIV/STI-testing history/health seeking behavior, v) sexual (risk) behaviors (gender differences), and vi) attitudes/behaviors toward people living with HIV.

### Methods

From October through December 2013, we conducted a cross-sectional survey on knowledge, attitudes, behaviors and practices on HIV, HEP and STI among MisSA living in Hamburg, Germany. Inclusion criteria for study participants were i) 18 years or older, ii) migrants from sub-Saharan Africa, iii) currently living in Hamburg.

We conducted this study as a community-based participatory research project, sharing the decision making power by researchers and community members [24]. The study design and the questionnaire were developed in an expert group of representatives from African communities, HIV/STI-prevention specialists and researchers. The details of this process as well as the development of study design are described elsewhere [25].

### Study site

The city of Hamburg has 1.8 million inhabitants, including approximately 13,000 officially registered MisSA [7]; this represents the largest sub-Saharan African community in Germany.

The “AIDS-Hilfe Hamburg e.V.” (AIDS-foundation Hamburg) (AHH) was chosen as a local partner organization, because AHH already had well-established contacts into different local African communities. Ethics approval was received from the ethics board at the Hamburg Chamber of Physicians (PV4553).

### Measurements

#### Questionnaire development

We included all essential indicators suggested by ECDC for behavioral surveillance in migrant populations [15]. As a draft for survey development we utilized a questionnaire successfully used in a KABP-survey on HIV in African migrants in the UK [19], and added questions on HEP and on local HIV testing services. We adapted the questionnaire after obtaining feedback from the expert group.

In order to determine comprehensibility, recall strategy, issues with sensitivity and social desirability of survey instrument, trained community members administered the questionnaire to five MisSA while simultaneously collecting additional verbal information on the responses and response options (cognitive interviewing) [26–28]. We then conducted pre-testing of the questionnaire with 35 African community members. Layout of the questionnaire and pre-test results were discussed with AHH and peer researchers resulting in removal of some questions on socio-demographic information and sexual attraction. African community members (two translators per language) translated the questionnaire into English and French. Independent native English and French speakers verified the translation.

The following sections were covered in the questionnaire: socio-demographic data, knowledge on HIV, HEP and STI, HIV/STI testing, sexual behavior and behavior toward people living with HIV. For more detail, see Table 1. Knowledge on HIV, HEP and STI was determined with “informing statements”. Participants were asked whether they were aware of specific information, (e.g. Did you know this before now? AIDS is caused by a virus called HIV.) The response options were “I knew this before”, “I did not know this”, “I am not sure if I knew this” and “I do not understand this”. Community partners supported this survey design, because study participants can learn and thus, benefit from completing the questionnaire. Throughout this paper we are using the term “knowledge gaps” when we are referring to lack of knowledge about HIV, HEP or STI.

**Table 1:** KABP-survey-sections and operationalization

Section	Items (I)
Socio-demographic and personal characteristics	Sex, age, country of birth, time in Germany, health insurance status, German language, level of education, religion, circumcision/genital mutilation,
Knowledge on Hepatitis B/C	(I1) Hepatitis is a disease of the liver caused by a virus.
*Use of “informing statements”*	(I2) Hepatitis B and C can be transmitted through blood or used needles.
*“Did you know this before now?”*	(I3) Hepatitis B can be transmitted through sexual contact.
	(I4) Hepatitis B can also be transmitted from mother to child.
	(I5) Chronic hepatitis can be treated successfully.
Knowledge on HIV	(I6) AIDS is caused by a virus called HIV.
*Use of “informing statements”*	(I7) You cannot tell from someone’s appearance whether he or she has HIV or not.
*“Did you know this before now?”*	
	(I8) There is a test which shows whether someone is HIV positive or not.
	(I9) Africans are NOT deported from Germany just for having HIV.
	(I10) In Hamburg, you can get tested for HIV- anonymously and for free.
	(I11) There is no cure for HIV infection.
	(I12) HIV and AIDS also exist in Germany.
	(I13) There are medications that can help people with HIV stay healthy.
	(I14) HIV is not transmitted through kissing or shaking hands.
	(I15) HIV can be transmitted through sexual intercourse.
Knowledge on STI	(I16) gonorrhoea, (I17) syphilis, (I18) herpes
*“Have you ever heard of these STI?”*	(I19) genital warts, (I20) chlamydia
HIV and STI testing	Ever tested for HIV or STI, testing without consent, STI diagnoses
Sexual behavioral risk	Sexual activity, sex and condom use with casual (non-steady) partners, reasons for not using condoms, number of sex partners, sexual violence
Behavior/attitudes toward PLWH	Personal connection, reactions towards PLWH (personal and within the community)

### Study population and data collection

#### Data collection

Data collection took place in Hamburg from October-December 2013 and was conducted by fourteen trained peer researchers who were recruited by the local study coordinator and other community organizations. Seven of fourteen peer researchers were female. Peer researchers’ age ranged from 18 to 45 years. Country of birth (e.g. Ghana, Togo, Sierra Leone, Cameroon, Mali, Burkina Faso, Benin, Somalia and Nigeria) reflected the most frequent of the officially registered MisSA-populations in Hamburg [7]. All peer researchers spoke German, English or French and different African languages and dialects. Before data collection, we conducted a training session on research ethics, recruitment strategy and basics on HIV, HEP and STI as well as a community mapping to identify potential recruitment sites (e.g. churches, Afro shops, stores, cultural and sport events). Within the period of data collection peer researchers determined recruitment times based on their availability and/or potential event schedules. Peer researchers approached African community members in the recruitment sites and obtained oral informed consent to participate in the survey.

Participants could choose between self-completion of written questionnaire in English, French or German or person-to person interview during which peer researchers provided translation into other African languages if needed. Study participants received a self-addressed stamped envelope for mailing questionnaires to Robert Koch Institute (RKI) directly. Recruited participants received a give-away consisting of a key chain with RKI logo, a shopping cart chip with an Africa map and a referral to “Café Africa” (a group for Africans where health promotion information sessions on HIV/STI are offered by AHH), a condom, as well as informational flyers on HIV, HEP, STI and the free testing services at the HIV/STI-voluntary testing and counselling site.

To minimize potential selection bias through peer researcher recruitment approach and to enhance representativeness of study population, we provided a weekly summary of recruited study population by sex, age, country of origin, language and level of education and encouraged peer researchers to approach certain under-reached groups.

We calculated a minimum sample size of 373, based on the number of officially registered MisSA in Hamburg (13,000), a confidence level of 95 %, and a confidence interval of 5 %.

### Data entry and statistical data analysis

We manually entered the questionnaire via VOXCO (an online survey and data collection software) into an SPSS database. Questionnaires were excluded if i) data on country of birth and/or sex was missing, ii) participants were not from sub-Saharan Africa, iii) participants were not living in Hamburg, iv) more than 40 % of information was missing. We included only complete cases (based on inclusion criteria) in our analysis.

Primary outcomes were knowledge on HIV (general and specific knowledge), HEP and STI, sexual behavior (sex differences), health seeking behavior (HIV/STI-testing history), sexual (risk) behavior and behavior toward PLWH. As explanatory variables (main predictors) we determined sex, level of education and German language skills, health insurance status, religion and age (Table 2).

**Table 2:** Presentation of objectives, outcome variables and conducted statistical analysis

Objective	Outcome variable(s)	Statistical analysis
Knowledge gaps on HIV	*General knowledge on HIV* (transmission and treatment):	- > multiple logistic regression
		- > binary outcome (I knew this information before or I did not know this before).
	- > aggregated number of positive responses (I knew this before) (items 6–8 and 11–15)	
	*Specific knowledge on HIV:*	- > predictors included in the model: sex, age group, school education, level of German language, health insurance, religion
	(testing services and deportation)	
	- > aggregated number of positive responses (I knew this before) (items 9–10)	
		- > adjusted for mode of administration
Knowledge gaps on HEP	*Knowledge on hepatitis:*	- > multiple logistic regression
	- > aggregated number of positive responses (I knew this before) (items 1–5).	- > binary outcome (I knew this information before or I did not know this before).
		- > predictors included in the model: sex, age group, school education, level of German language, health insurance, religion
		- > adjusted for mode of administration
Knowledge gaps on STI	*Knowledge of STI:*	- > multiple logistic regression
	- > aggregated number of positive responses on five STI (items 16–20); all responses “I have heard of this STI before” versus “I had not heard of this before”.	- > binary outcome (I knew of STI or did not know of STI before).
		- > predictors included in the model:
		sex, age group, school education, level of German language, health insurance, religion
		- > adjusted for mode of administration
Health seeking behavior	- > Ever tested for HIV	- > descriptive analysis stratified by sex, age group, school education, religion
	- > Ever tested for an STI	
	- > Ever diagnosed with an STI	- > unadjusted odds ratios
Sexual (risk) behavior in the last 12 months	- > Number of partners	- > descriptive analysis stratified by sex, age group, school education, religion
	- > Steady partnership	
	- > Steady partnership with German native	
		- > unadjusted odds ratios
	- > Sex with casual partners	
	- > Number of sex partners	
	- > Condom use with casual partners	
	- > Reasons for not using condoms	
	- > Experience of sexual violence (ever)	
Attitudes and behavior toward PLWH	- > Personally knowing someone with HIV.	- > descriptive analysis stratified by sex, school education
	- > treatment of PLWH in the community	- > unadjusted odds ratios
	- > Behavior toward PLWH	

#### Analysis regarding knowledge on HIV, HEP, STI

First, we performed a descriptive analysis of all knowledge items (HIV, HEP, STI). To determine potential differences on knowledge by basic demographic information such as gender, age, religion, time living in Germany, level of education or health insurance status, we stratified for these categories. We additionally stratified by mode of survey completion to control for mode of administration bias. All significant results from this analysis can be found in Additional file 1.

Secondly, we summarized items on knowledge by infection and/or based on results from univariate analysis, resulting in four categories: general knowledge on HIV transmission and treatment (I6-8, I11-15); specific knowledge on HIV (I9-10), knowledge on hepatitis (I1-5) and knowledge on STI (I16-20). Specific knowledge on HIV included two items that were addressing local knowledge on the situation in Germany (HIV-testing services in Hamburg and deportation from Germany).

Multiple logistic regression analysis was conducted to identify factors independently associated with knowledge on HIV, HEP and STI using the aggregated number of positive responses (“I knew this before”) per section. The respective adjusted odds ratios were calculated. We included all factors found to differ among sub-groups in stratified analysis using a significance level of p ≤ 0.05 of all results in univariate analyses and adjusted for mode of questionnaire administration. We controlled for interactions between time living in Germany and German language skills as well as education and level of German language (Table 2).

#### Analysis regarding sexual behavior, HIV/STI-testing history and behavior toward PLWH

Indicators on sexual behavior, HIV/STI-testing history and behavior toward PLWH were stratified by sex, level of education, age group and religion (Table 2). Complete results of analysis can be found in Additional file 2. To detect differences between groups, chi-square test and unadjusted odds ratios (OR) including 95 % confidence interval (95 % CI) were calculated.

Data was analyzed using SPSS 20.0 and Stata 13.

## Results

### Description of study population

Of a total of 950 questionnaires that were distributed to potential participants, 649 arrived at RKI and 569 met the inclusion criteria. Most common mode of survey administration was self-completion (49 %), followed by face-to-face (38 %) and telephone interviews (13 %) (Table 3).

**Table 3:** Socio-demographic characteristics of study population

	Men	Women	Total
Participants	326 (57.3 %)	243 (42.7 %)	569 (100.0 %)
Age groups (n = 549)			
18 – 25 years	78 (24.9 %)	67 (28.4 %)	145 (26.4 %)
26 – 35 years	122 (39.0 %)	98 (41.5 %)	220 (40.1 %)
36 – 45 years	83 (26.5 %)	59 (25.0 %)	142 (25.9 %)
≥45 years	30 (9.6 %)	12 (5.1 %)	42 (7.7 %)
Median (Min, Max)	31 (18–70)	30 (18–55)	30 (18–70)
Region of birth (n = 568)			
Western Africa	262 (80.6 %)	163 (67.1 %)	425 (74.8 %)
Central Africa	39 (12.0 %)	53 (21.8 %)	92 (16.2 %)
Eastern Africa	19 (5.8 %)	23 (9.5 %)	42 (7.4 %)
Southern Africa	5 (1.5 %)	4 (1.6 %)	9 (1.6 %)
Country of birth, Top 3			
Ghana	44 (13.5 %)	41 (16.9 %)	85 (14.9 %)
Cameroon	30 (9.2 %)	42 (17.3 %)	72(12.7 %)
Togo	33 (10.1 %)	29 (11.9 %)	62 (10.9 %)
Time in Germany (n = 565)			
<1 year	32 (9.9 %)	11 (4.5 %)	43 (7.6 %)
1 - < 5 years	114 (35.4 %)	82 (33.7 %)	196 (34.7 %)
5 - < 10 years	64 (19.9 %)	82 (33.7 %)	146 (25.8 %)
10 - <20 years	96 (29.8 %)	57 (23.5 %)	153 (27.1 %)
≥20 years	16 (5.0 %)	11 (4.5 %)	27 (4.8 %)
Median (Min, Max)	6 years (1 m-41 y)	6 years (1 m-41 y)	6 (1 m-41 y)
Level of education (n = 566)			
Primary/Secondary School	109 (33.6 %)	83 (34.3 %)	192 (33.9 %)
High School/Vocational School	89 (27.5 %)	77 (31.8 %)	166 (29.3 %)
University/College	98 (30.2 %)	63 (26.0 %)	161 (28.4 %)
No certificate	27 (8.3 %)	18 (7.4 %)	45 (8.0 %)
Other	1 (0.3 %)	1 (0.4 %)	2 (0.4 %)
Level of German language (n = 560)			
Very good	65 (20.2 %)	36 (15.1 %)	101 (18.0 %)
Good	106 (33.0 %)	95 (39.7 %)	201 (35.9 %)
Satisfactory	85 (26.5 %)	76 (31.8 %)	161 (28.7 %)
Little	40 (12.5 %)	20 (8.4 %)	60 (10.7 %)
None	25 (7.8 %)	12 (5.0 %)	37 (6.6 %)
Health insurance in Germany (n = 537)			
Yes	241 (78.0 %)	202 (88.6 %)	443 (82.5 %)
No	68 (22.0 %)	26 (11.4 %)	94 (17.5 %)
Religion (n = 564)*			
Christianity	152 (46.9 %)	174 (72.5 %)	326 (57.8 %)
Islam	146 (45.1 %)	47 (19.6 %)	193 (34.2 %)
Traditional African Religion	6 (1.9 %)	11 (4.6 %)	17(3.0 %)
Other	1 (0.3 %)	4 (1.7 %)	4 (0,9 %)
No religion	22 (6.8 %)	12 (5.0 %)	34 (6.0 %)
Steady relationship (n = 541)			
Yes	158 (51.3 %)	164 (70.4 %)	322 (69.5 %)
No	150 (48.7 %)	69 (29.6 %)	219 (40.5 %)
Circumcision/genital mutilation (n = 544)			
Yes	278 (88.8 %)	69 (29.9 %)	347 (63.8 %)
No	35 (11.2 %)	162 (70.1 %)	197 (36.2 %)

*multiple responses were possible

A total of 43 % of participants were women and 57 % were men. The median age was 31 years for men (range = 18–70) and 30 years for women (range = 18-55). The majority of participants came from countries in Western (75 %) and Central (16 %) sub-Saharan Africa. Most common countries of birth were Ghana (15 %), Cameroon (13 %) and Togo (11 %) (Table 3). Median time of residence in Germany was 6 years in men and women. The majority of participants (65 %) reported satisfactory or good level of German language, whereas 20 % of men and 13 % of women stated little or no German language skills (Table 3). A total of 34 % had a primary or secondary school degree, 29 % finished high school or vocational school and 28 % had a university degree. The majority of study population was Christian (57 %), followed by Muslim (34 %) and no religion (6 %). However, women more often were Christian (72 %) than men (47 %) (p < 0.001). 51 % of men and 70 % of women reported to be in a steady relationship (p < 0.001).

Most men were circumcised (89 %), regardless of religious affiliation (Muslims = 91 % and Christians 89 %). 30 % of women reported genital mutilation. There were no significant differences in prevalence of genital mutilation, age or time in Germany (Table 3).

### Access to health care

Overall, 83 % of participants reported to have health insurance in Germany. Women were more often insured than men (89 % vs. 78 %; p = 0.001). The majority of participants (78 %) went to see a doctor within the last 12 months, with women reporting more often access to health care than men (92 % vs. 77 %; p < 0.001). When experiencing medical problems 77 % of participants reported to go to a doctor and 36 % to the hospital. Some participants ask friends for help (6 %) and some go to see an African healer (4 %). Men more often did not know where to seek care (6 % vs. 2 %; p = 0.02).

### Knowledge about HIV, hepatitis and STI and testing

#### Knowledge about HIV

The level of knowledge on HIV was higher than on viral hepatitis. Eight of ten statements were known by more than 80 % of participants. A total of 56 % of respondents were confident that Africans will not be deported from Germany just for having HIV and 36 % were familiar with the anonymous and free HIV/STI-testing service in Hamburg; women were less aware of HIV-testing services than men (30 % vs. 42 %; OR = 1.7; 95 % CI [0.4-0.8]) and persons with limited German language skills knew this information less often than persons with intermediate or higher levels of German (29 % vs. 43 %; OR = 1.8; 95 % CI [1.3-2.6]).

Variables that had an impact on overall general knowledge about HIV were sex, age, school education, level of German language, health insurance status and religious affiliation. In univariate analysis female sex and higher levels of education were associated with better overall knowledge. Participants who indicated low levels of German language or Islam as religion more often did not know presented information. Higher level of German language was positively associated with knowledge on HIV. In multivariate analysis high levels of German language was associated with higher levels of overall knowledge on general information on HIV, whereas not having health insurance in Germany was associated with less knowledge on the presented statements (Table 4).

**Table 4:** Factors associated with knowledge on HIV, HEP and STI: Results from univariate and multivariate analysis *(aggregated number of positive responses (I knew this before) per section)*

Knowledge on HIV transmission risks and treatment (I6-8, I11-15)**(n = 521)								
		Proportion	Univariate analysis			Multivariate analysis		
Variable	Characteristic	%	OR	p-value	95 % CI	OR	p-value	95 % CI
Sex	Men	91.4	1.0			1.0		
	Women	94.8	1.7	<0.001	1.3-2.21	1.5	0.12	0.9-2.5
Age group	18-25	89.5	0.6	<0.001	0.4-0.74	0.6	0.14	0.4-1.2
	26-35	93.8	1.0			1.0		
	36-45	95.0	1.3	0.18	0.9-1.8	1.1	0.80	0.6-2.0
	>45	96.4	1.8	0.07	0.96-3.3	2.0	0.20	0.7-5.8
School education	No degree/certificate	89.3	0.8	0.28	0.6-1.2	0.9	0.85	0.4-2.1
	Primary/secondary School	91.2	1			1.0		
	High -/vocational school	93.4	1.4	0.03	1.0-1.8	1.4	0.22	0.8-2.6
	University degree	95.8	2.2	<0.001	1.6-3.1	2.1	0.03	1.1-4.1
Level of German language	Very good	96.8	1.7	0.01	1.1-2.6	2.9	0.02	1.2-6.8
	Good	96.4	1.0			1.0		
	Satisfactory	93.9	0.9	0.36	0.7-1.2	0.8	0.44	0.4-1.4
	Little	87.3	0.5	<0.001	0.3-0.6	0.7	0.47	0.3-1.6
	None	85.5	0.4	<0.001	0.3-0.6	1.1	0.90	0.4-2.8
Health insurance	Yes	85.7	1.0			1.0		
	no	94.8	0.3	<0.001	0.3-0.4	0.3	<0.001	0.2-0.5
Religion	Christianity	95.3	1.0			1.0		
	Islam	88.7	0.4	<0.001	0.3-0.5	0.6	0.07	0.4-1.1
	No religion	95.9	1.2	0.49	0.6-2.2	1.7	0.38	0.5-5.2
Specific knowledge on HIV (I9-10)**(n = 520)								
		Proportion	Univariate analysis			Multivariate analysis		
Variable	Characteristic	%	OR	p-value	95 % CI	OR	p-value	95 % CI
Sex	Men	48.8	1.0			1.0		
	Women	42.8	0.8	0.05	0.6-1.0	0.7	0.05	0.5-0.997
Age group	18-25	38.0	0.7	0.04	0.5-0.99	0.7	0.07	0.5-1.0
	26-35	45.6	1.0			1.0		
	36-45	51.6	1.3	0.12	0.9-1.7	1.3	0.24	0.9-1.8
	>45	58.5	1.7	0.03	1.0-2.7	1.5	0.19	0.8-2.7
School education	No degree/certificate	41.1	0.9	0.71	0.6-1.5	1.1	0.80	0.6-1.9
	Primary/secondary School	43.3	1.0			1.0		
	High -/vocational school	42.6	1.0	0.85	0.7-1.3	0.8	0.25	0.5-1.2
	University degree	55.1	1.6	0.002	1.2-2.2	1.2	0.30	0.8-1.8
Level of German language	Very good	67.5	2.3	<0.001	1.6-3.2	2.2	<0.001	1.4-3.5
	Good	48.0	1.0			1.0		
	Satisfactory	40.8	0.8	0.06	0.6-1.0	0.8	0.29	0.6-1.2
	Little	26.9	0.4	<0.001	0.3-0.6	0.3	<0.001	0.2-0.6
	None	30.6	0.5	0.01	0.3-0.8	0.4	0.01	0.2-0.8
Health insurance	Yes	45.2	1.0			1.0		
	no	46.5	1.0	0.71	0.7-1.3	1.4	0.14	0.9-2.1
Religion	Christianity	48.7	1.0			1.0		
	Islam	41.8	0.8	0.03	0.6-0.98	0.7	0.04	0.5-0.98
	No religion	57.6	1.4	0.17	0.9-2.4	1.4	0.34	0.7-2.5
Knowledge on hepatitis (I1-5)**(n = 519)								
		Proportion	Univariate analysis			Multivariate analysis		
Variable	Characteristic	%	OR	p-value	95 % CI	OR	p-value	95 % CI
Sex	Men	50.8	1			1.0		
	Women	49.1	0.9	0.37	0.8-1.1	0.8	0.40	0.5-1.3
Age group	18-25	45.2	0.8	0.003	0.6-0.9	0.6	0.15	0.4-1.2
	26-35	52.4	1.0			1.0		
	36-45	49.6	0.9	0.26	0.7-1.1	1.0	0.92	0.5-1.7
	>45	54.8	1.1	0.52	0.8-1.5	1.2	0.67	0.5-3.0
School education	No degree/certificate	16.1	0.3	<0.001	0.2-0.5	0.2	0.001	0.1-0.5
	Primary/secondary School	37.2	1			1.0		
	High -/vocational school	54.1	2.0	<0.001	1.6-2.4	2.9	<0.001	1.6-5.1
	University degree	71.7	4.3	<0.001	3.5-5.2	8,5	<0.001	4.5-15.9
Level of German language	Very good	71.3	2.5	<0.000	2.0-3.2	3.2	0.001	1.6-6.3
	Good	49.6	1			1.0		
	Satisfactory	46.3	0.9	0.17	0.7-1.1	0.8	0.54	0.5-1.5
	Little	40.3	0.7	0.005	0.5-0.9	0.6	0.29	0.3-1.5
	None	24.6	0.3	<0.001	0.2-0.5	0.4	0.096	0.1-1.2
Health insurance	Yes	51.9	1			1.0		
	No	43.2	0.7	<0.001	0.6-0.9	0.8	0.56	0.4-1.6
Religion	Christianity	55.6	1			1.0		
	Islam	38.6	0.5	<0.001	0.4-0.6	0.6	0.03	0.3-0.9
	No religion	64.5	1.5	0.03	1.1-2.0	2.1	0.12	0.8-5.7
Knowledge on STI (I16-20)**(n = 470)								
		Proportion	Univariate analysis			Multivariate analysis		
Variable	Characteristic	%	OR	p-value	95 % CI	OR	p-value	95 % CI
Sex	Men	44.4	1.0			1.0		
	Women	53.7	1.5	<0.001	1.2-1.7	1.4	<0.001	1.2-1.7
Age group	18-25	49.6	1.0	0.67	0.9-1.3	1.1	0.40	0.9-1.4
	26-35	48.5	1.0			1.0		
	36-45	48.1	1.0	0.88	0.8-1.2	1.1	0.64	0.8-1.3
	>45	47.8	1.0	0.85	0.7-1.3	1.1	0.56	0.8-1.5
School education	No degree/certificate	39.3	0.9	0.63	0.6-1.3	0.8	0.42	0.6-1.3
	Primary/secondary School	41.4	1.0			1.0		
	High -/vocational school	50.8	1.5	<0.001	1.2-1.8	1.5	0.001	1.2-1.8
	University degree	56.3	1.8	<0.001	1.5-2.2	1.9	<0.001	1.5-2.4
Level of German language	Very good	54.8	1.4	0.004	1.1-1.7	1.3	0.05	0.996-1.6
	Good	46.6	1.0			1.0		
	Satisfactory	49.6	1.1	0.19	0.9-1.4	1.1	0.43	0.9-1.3
	Little	41.1	0.8	0.11	0.6-1.1	1.1	0.58	0.8-1.5
	None	41.7	0.8	0.31	0.6-1.2	1.4	0.12	0.9-2.2
Health insurance	Yes	51.1	1.0			1.0		
	No	38.4	0.6	<0.001	0.5-0.7	0.6	<0.001	0.5-0.8
Religion	Christianity	50.9	1.0			1.0		
	Islam	40.5	0.7	<0.001	0.6-0.8	0.9	0.16	0.7-1.1
	No religion	65.3	1.8	<0.001	1.3-2.5	2.1	<0.001	1.4-2.96

**adjusted for mode of questionnaire administration

Higher level of “specific” knowledge on HIV was associated with sex, age, level of education and German language and religion. In univariate analysis being female or under 26 years of age, low levels of German language and Islam as religion were associated with lower levels of knowledge. Persons with university degree and very good German language skills more often reported knowing the presented information. In multivariate analysis low levels of German language and being Muslim were associated with lower levels of “specific” knowledge on HIV (Table 4).

#### Knowledge about hepatitis B and C

The proportion of participants knowing the presented information about viral hepatitis varied between 58 % (I1) and 40 % (I5). There were no gender differences in knowledge on hepatitis and women and men only differed in knowledge on treatment of chronic hepatitis (34 % vs. 45 %; 95 % CI [1.1-2.2]).

Variables that had an impact on overall knowledge about hepatitis were school education and religious affiliation. In univariate analysis, higher levels of education were associated with better overall knowledge. Participants who indicated Islam as religion more often did not know presented information, whereas persons with “no religion” had the highest levels of knowledge. Higher level of German language was positively associated with knowledge on HBV and HCV. In multivariate analysis lower level of education and German language as well as indicating Muslim as religious affiliation was associated with less knowledge on the presented statements (Table 4).

#### Knowledge about STI

Most participants had heard of syphilis (69 %) or gonorrhea (62 %) before, whereas herpes (33 %), genital warts (29 %) and chlamydia (28 %) were less known. A total of 13 % of all male participants and 6 % of all females had never heard of any listed STI. Women had more often heard of chlamydia (45 % vs. 20 %; OR = 3.3; 95 % CI [1.4-3.3]) and syphilis than men (83 % vs. 70 %; OR = 2; 95 % CI [2.0-5.0]).

In univariate analysis, female sex, higher level of education and German language, and having no religion were associated positively with high levels of overall knowledge about STI, whereas persons who did not have health insurance or were Muslim less often knew the presented information. In multivariate analysis, being female, high levels of education, and having no religious affiliation was associated with higher levels of overall knowledge on general information on STI, whereas the lack of a health insurance in Germany was associated with less knowledge on the presented statements (Table 4).

#### HIV and STI-testing

Overall, 67 % of all participants had ever been tested for HIV. Women more frequently reported a previous HIV-test than men (74 % vs. 63 %). Most respondents were tested within the last five years (76 %). Twenty-nine percent of those who had an HIV-test in the past were tested without consent when applying for asylum (47 %), at the hospital (31 %) or during pregnancy (28 %).

Of all persons (n = 489) who had heard of at least one STI before, 42 % of women and 34 % of men were ever tested for an STI with no statistical difference. However, men were more often diagnosed with an STI than women (58 % vs. 39 %) (Table 5).

**Table 5:** HIV/STI-testing history by sex

Sex	Men	Women	OR	p-value	95 %-CI
Ever tested for HIV	62.5 % (n = 202)	73.6 % (n = 178)	0.6	0.006	0.4–0.9
Ever tested for an STI	33.7 % (n = 89)	41.6 % (n = 92)	0.7	0.073	0.5–1.1
Ever diagnosed with an STI	56.2 % (n = 45)	38.8 % (n = 26)	2.0	0.035	1.1–3.3

### Sexual (risk) behavior

A total of 96 % of all participants reported that they ever had sex, and of those 88 % of men and 91 % of women had sex within the last 12 months. Women more often had a single sex partner than men and were more often in a steady relationship, whereas men reported twice as often (43 % vs. 23 %) having casual sex partners than women (Table 6). Also, men more frequently had more than 5 sex partners within the last 12 months and more often reported having a steady partner from Germany (42 % vs. 23 %) than women. Inconsistent condom use with non-steady partners was reported by 39 % of women and 33 % of males. Self-reported prevalence of sexual violence was higher in women than in men (16 % vs. 6 %) (Table 6). Being over 30 years of age was associated with having a single sex partner, less frequent sex with casual partners and a lower percentage of steady partners from Germany (Additional file 2).

**Table 6:** Gender differences in sexual behavior and reasons for not using condoms

Sexual activity	Men	Women	OR	p-value	95 %-CI
Ever had sex	95.7 % (n = 291)	96.0 % (n = 216)	0.9	0.88	0.39–2.2
Sex within the last 12 months	88.4 % (n = 267)	90.9 % (n = 210)	0.8	0.351	0.4–1.3
Single sex partner (12 months)	47.9 % (n = 126)	73.4 % (n = 152)	0.3	0.000	0.2–0.5
More than 5 sex partners (12 months)	4.6 % (n = 12)	1.9 % (n = 4)	2.4	0.118	0.8–7.6
Steady sexual partner(s)	67.9 % (n = 178)	79.9 % (n = 163)	0.5	0.004	0.3–0.8
Steady sexual partner(s) from Germany	41.7 % (n = 73)	23.9 % (n = 39)	2.3	0.001	1.4–3.6
Sex with casual partner(s)	43.2 % (n = 99)	22.7 % (n = 41)	2.6	0.000	1.7–4.0
Inconsistent condom use with casual partners	33.0 % (n = 32)	38.5 % (n = 15)	0.8	0.544	0.4–1.7
Sexual violence (once and/or repeated)	6.1 % (n = 19)	15.8 % (n = 37)	0.3	0.000	0.2–0.6
Reasons for the not using condoms* n = 480	Men	Women	OR	p-value	95 %-CI
They are expensive	3.3 % (n = 9)	1.4 % (n = 3)	2.3	0.199	0.6–8.7
I don’t know where to get them.	2.9 % (n = 8)	2.4 % (n = 5)	1.2	0.731	0.4–3.8
I find it embarrassing.	9.2 % (n = 25)	12.1 % (n = 25)	0.7	0.300	0.4–1.3
I don’t want anybody to see me buying condoms.	5.1 % (n = 14)	6.3 % (n = 13)	0.8	0.294	0.4–1.8
I would like to get pregnant/have a child with my partner.	13.6 % (n = 37)	30.0 % (n = 62)	0.4	<0.000	0.2–0.6
My partner might think I have other sex partners or might have HIV.	10.6 % (n = 29)	9.2 % (n = 19)	1.2	0.601	0.6–2.2
My partner doesn’t want to use it.	15.4 % (n = 42)	20.3 % (n = 42)	0.7	0.161	0.4–1.1
Because of my religion.	4.0 % (n = 11)	2.4 % (n = 5)	1.7	0.329	0.6–5.0
I don’t like condoms.	12.1 % (n = 33)	13.5 % (n = 28)	0.9	0.639	0.5–1.5
I want to feel my partner.	22 % (n = 59)	14 % (n = 29)	1.7	0.033	1.0–2.8
I am monogamous.	9.5 % (n = 26)	10.1 % (n = 21)	0.9	0.821	0.5–1.7
I don’t have sex.	2.2 % (n = 6)	2.4 % (n = 5)	0.9	0.875	0.3–3.0
Other reasons	5.9 % (n = 16)	8.7 % (n = 18)	0.7	0.231	0.3–1.3
I always use a condom.	32.2 % (n = 88)	28.0 % (n = 58)	1.2	0.320	0.8–1.8

*multiple response options

There were differences in reasons for not using condoms between women and men. Women more often reported that they wanted to have a child, and men more frequently indicated that they wanted to feel their partner. Other common reasons were the sex partner’s rejection of condoms, fear of being seen when purchasing condoms, monogamy, embarrassment and that partner might assume an HIV infection or multiple sex partners (Table 6). There were no relevant differences in sexual behavior by education or religious affiliation (Additional file 2).

### Attitudes and behavior towards PLWH

Of all participants 39 % of men and 42 % of women reported to personally know someone living with HIV. Forty percent indicated that they had heard of PLWH in their community being treated badly, and 65 % agreed that HIV was a topic that is talked about within the respective communities.

The majority of respondents (75 %) reported that they would treat PLWH like any other person. Some respondents indicated avoiding physical contact, being seen with someone with HIV or that they blame that person secretly for their serostatus. Knowing a PLWH personally was not associated with different behavior toward them. Women and people with higher levels of education more often reported treating a PLWH like any other person. Conversely, participants with lower education more frequently reported avoiding physical contact or being seen with PLWH (Table 7).

**Table 7:** Attitudes and behavior toward PLWH by sex, education

Behavior toward PLWH	Men	Women	OR	p-value	95 %-CI
I treat them like any other person.	72.1 % (n = 209)	79.6 % (n = 183)	0.7	0.049	0.4–1.0
I avoid physical contact.	12.8 % (n = 37)	10.9 % (n = 25)	1.2	0.509	0.7–2.1
I avoid being seen with this person.	7.2 % (n = 21)	5.2 % (n = 12)	1.4	0.347	0.7–3.0
I blame this person secretly.	9.0 % (n = 26)	11.7 % (n = 27)	0.7	0.299	0.4–1.3
I behave differently.	5.2 % (n = 15)	3.9 % (n = 9)	1.3	0.497	0.6–3.1
Education	Education ↓	Education ↑	OR	p-value	95 %-CI
I treat them like any other person.	79.2 % (n = 154)	71.3 % (n = 236)	0.7	0.039	0.4–0.9
I avoid physical contact.	8.7 % (n = 26)	16.2 % (n = 35)	2.0	0.010	1.2–3.5
I avoid being seen with this person.	4.4 % (n = 13)	8.3 % (n = 18)	2.0	0.062	1.0–4.2
I blame this person secretly.	8.4 % (n = 25)	12.5 % (n = 27)	1.6	0.127	0.9–2.8
I behave differently.	4.7 % (n = 14)	4.2 % (n = 9)	0.9	0.774	0.4–2.1
Age	≤ 30 years old	> 30 years old	OR	p-value	95 %-CI
I treat them like any other person.	70.1 % (n = 169)	80.7 % (n = 209)	0.6	0.006	0.4–0.9
I avoid physical contact.	13.3 % (n = 32)	10.4 % (n = 27)	1.3	0.323	0.8–2.3
I avoid being seen with this person.	8.7 % (n = 21)	3.5 % (n = 9)	2.7	0.014	1.2–5.9
I blame this person secretly.	8.7 % (n = 21)	11.6 % (n = 30)	0.7	0.289	0.4–1.3
I behave differently.	4.6 % (n = 11)	5.0 % (n = 13)	0.9	0.812	0.4–2.1
Religion	Christianity	Islam	OR	p-value	95 %-CI
I treat them like any other person.	76.9 % (n = 223)	71.4 % (n = 120)	1.3	0.193	0.9–2.1
I avoid physical contact.	10.7 % (n = 31)	14.3 % (n = 24)	0.7	0.254	0.4–1.3
I avoid being seen with this person.	5.2 % (n = 15)	7.7 % (n = 13)	0.7	0.269	0.3–1.4
I blame this person secretly.	10.7 % (n = 31)	10.1 % (n = 17)	1.1	0.848	0.6–2.0
I behave differently.	4.8 % (n = 14)	4.8 % (n = 8)	1.0	0.975	0.4–2.5

## Discussion

We conducted a KABP-survey among MisSA to identify knowledge gaps, health seeking and sexual risk behavior as well as attitudes toward PLWH. Knowledge about HIV transmission and treatment was generally good, whereas study participants demonstrated knowledge gaps regarding viral hepatitis, STI and testing services. Some sub-groups, e.g. persons with limited German language skills, lower education, without health insurance or of Muslim faith had more pronounced knowledge gaps. Men had more sex partners than women and more often reported a previous STI-diagnosis, whereas experience of sexual violence was more common among women.

### Knowledge gaps and information needs

The study population was well informed about general aspects of HIV/AIDS, such as transmission risks. These results are in line with other European studies targeting African community members [19, 23, 29, 30] and might indicate that HIV prevention efforts over the last decades have been successful, especially after the United Nations Millennium Declaration in 2000 set the goal of reversing the HIV-epidemic by 2015, with a focus on sub-Saharan African countries [31–33]. Our results show that knowledge about free HIV/STI-testing services was low (just one third of study population knew this) and the fact that HIV diagnoses do not lead to deportation was only known by half of participants. This information is specific to Germany and shows the importance of providing information about the local health care systems to African communities, especially to persons with limited German language skills and lower levels of education. Furthermore, fear of deportation may constitute a barrier to HIV-testing and care [10, 34] and should thus be addressed in prevention messages.

There was limited knowledge on viral hepatitis as well as on STI, which until recently have not been addressed in national prevention efforts as much as HIV. Given the impact of migration on the epidemiology of HEP and potentially STI [3], this calls for a further expansion on prevention activities to include HEP and STI. Here, we can use the experiences and examples set by global HIV prevention measures to comprehensively address these topics [31, 32, 35]. In primary prevention efforts for African communities in Germany it is particularly important to provide multi-lingual materials in order to reach people with limited German skills.

Also, given the more pronounced need for information, we recommend targeting health promotion activities for Muslim communities. Other research shows that in comparison to other religious groups, Muslims had lower levels of knowledge regarding HIV and STI [19, 23], potentially due to a lack of HIV prevention in Muslim countries. [36, 37].

### Considerations for prevention planning

HIV-transmissions within African communities in Europe are not uncommon and potentially underestimated [8] but the background prevalence in country of birth is most likely higher[31]. Thus, the likelihood of being initially diagnosed with infectious diseases might be higher in recent migrants. For prevention planning this highlights the importance of assuring access to HIV-testing and care for all, but specifically for newly arrived migrants [10, 38]. Also, information on potentially higher background prevalence in country of birth and its implications for infection risks when visiting friends and relatives should be included in prevention messages [39, 40].

The majority of participants had seen a physician within the last 12 months. Considering this, it seems crucial to involve general practitioners in prevention planning. However, studies have shown that clinicians often fail to address HIV-prevention even with their seropositive patients [41, 42]. Thus, training of physicians is important to assure culturally sensitive care, especially for those who are well-known in African communities.

Our results show that level of German language was positively associated with knowledge on HIV, HEP and STI. This association might be partially explained by an enhanced comprehension of survey questionnaires by participants with high levels of German; if survey was completed in German. Participants with high levels of German language might also have better access to German language prevention programs.

### Attitudes and behavior toward PLWH

Fear of potential stigmatization of PLWH by people from the African communities as well as health care providers is an often cited barrier to accessing testing and care [3, 10, 43]. In our study, the majority of participants intended to treat PLWH like any other person. However, less than half of respondents personally knew someone with HIV and thus, this level of acceptance might be biased by social desirability.

### A need for gender-sensitive prevention

Men in our study report higher prevalence of casual sex and more sex partners. Moreover, the reported STI diagnoses are higher in men, which might indicate higher infection risks for men due to higher partner numbers or lower probability of STI becoming symptomatic and being subsequently diagnosed in women. Women report higher prevalence of sexual violence and lower number of casual partners. Thus, specific prevention topics for women should include empowerment to use condoms and negotiate condom use, and addressing sexual violence. Other European studies support these prevention messages: African women were more likely to have difficulties communicating about safer sex and HIV with their sex partners [19, 23, 44]. The same studies showed that men were less likely to be concerned about HIV transmission than women and more often engaged in sexual behavior with risk for infection [19, 23]. Hence, the conversation about risk-assessment and promoting gender equality are important prevention messages for men. Further research should investigate the roots and underlying factors of male and female risk behavior with qualitative methods.

### Limitations

There are some limitations to consider when interpreting our results. We used convenience sampling and thus, cannot be sure, that our sample was representative for the MisSA population in Hamburg. Even though we used targeted recruitment, based on population statistics, to limit these affects, these statistics did not include MisSA without legal status, or persons who were naturalized and accepted German citizenship.

There may have been a social desirability bias in the answers of the respondents, especially when reporting the degree of condom use or talking about sexual practices. Also, mode of administration might have influenced responses, even though we controlled for that in analysis.

Underlying social norms and ideas of gender roles, such as masculinity might have caused underreporting of sexual activity in women and/or over-reporting of sexual activity among men [36, 45, 46]. Also, sexual violence might have been underreported due to stigmatization and shame or fear of other undesired consequences.

The use of “true statements” for questions on knowledge might have caused overly positive results, and thus, overestimated true knowledge of participants. This has to be taken into account when interpreting data and might indicate that knowledge gaps are higher than expected. We tried to minimize this effect by dichotomizing the answer options and only accepting explicit knowledge as knowledge.

The questionnaire was not offered in any African languages. This decision was based on feedback from African community partners who considered translation into English and French sufficient if peer researchers spoke the most common African languages.

Lastly, the sample was too small to see differences in knowledge and behavior in different regions of origin. Also, the generated knowledge might be specific to Hamburg. Because of that we are currently planning a multi-center study in five different regions in Germany. With an expected sample size of 3,000 (including the sample in Hamburg) a more detailed analysis will be possible.

## Conclusions

With this study, we showed that a community-based participatory research approach can be utilized in KABP-surveys on HIV, HEP and STI. We observed good overall knowledge on HIV and found substantial information gaps on HEP and STI. Given the more pronounced information gaps, we recommend targeting health promotion and prevention activities for people with limited German language skills and in Muslim communities. Men are having more sex partners than women, whereas women are reporting more sexual violence and thus are demonstrating different prevention needs and risks for acquiring HIV, HEP or STI. This calls for gender-sensitive prevention efforts in African communities in Germany.

## Additional files

Additional file 1:
**Stratified analysis of items on knowledge about HIV, HEP and STI (participants who responded “I knew this already”) by basic demographic information, partnership status and mode of administration.** Significant differences only (p<0.05). (DOCX 47 kb) Additional file 2:
**Results of stratified analysis of sexual behavior by school education, age group and religion, HIV/STI-testing history by school education, age and religion and attitudes toward PLWH by age and religion.** (DOCX 31 kb)
