# Supplementary material for: Determinants of HIV, viral hepatitis and STI prevention needs among African migrants in Germany; a cross-sectional survey on knowledge, attitudes, behaviors and practices
Source: BMC Public Health. 2015 Aug 6;15:753. doi: 10.1186/s12889-015-2098-2 (PMC4545823; doi:10.1186/s12889-015-2098-2)
Supplement: Additional file 1: — Stratified analysis of items on knowledge about HIV, HEP and STI (participants who responded “I knew this already”) by basic demographic information, partnership status and mode of administration. Significant differences only (p<0.05). (DOCX 47 kb) [file 12889_2015_2098_MOESM1_ESM.docx]

**Additional File 1**

Stratified analysis of items on knowledge about HIV, HEP and STI (participants who responded „I knew this already“) by basic demographic information, partnership status and mode of administration. Significant differences only (p<0.05)

Table S1: Knowledge on hepatitis (participants who responded „I knew this already“) by basic demographic information, partnership status and mode of administration. Significant differences only (p<0.05)*

| **Sex** | **Men** | **Women** | **OR** | **p-value** | **95%-CI** |
| --- | --- | --- | --- | --- | --- |
| Chronic Hepatitis can be treated successfully. | 45% (n=143) | 34% (n=81) | 1.6 | 0.010 | 1.1-2.2 |
| **Religion** | **Christianity** | **Islam** | **OR** | **p-value** | **95%-CI** |
| Hepatitis is a disease of the liver caused by a virus. | 64% (n=207) | 46% (n=87) | 2.1 | 0.000 | 1.4-3.0 |
| Hepatitis B and C can be transmitted through blood or used needles. | 62% (n=199) | 38% (n=72) | 2.6 | 0.000 | 1.8-3.7 |
| Hepatitis B can be transmitted through sexual contact. | 61% (n=199) | 40% (n=75) | 2.4 | 0.000 | 1.7-3.5 |
| Hepatitis B can also be transmitted from mother to child. | 49% (n=158) | 33% (n=63) | 1.9 | 0.001 | 1.3-2.8 |
| **Education ^1^** | **Education ↓** | **Education ↑** | **OR** | **p-value** | **95%-CI** |
| Hepatitis is a disease of the liver caused by a virus. | 40% (n=94) | 72% (n=233) | 0.3 | 0.000 | 0.2-0.4 |
| Hepatitis B and C can be transmitted through blood or used needles. | 35% (n=82) | 68% (n=219) | 0.3 | 0.000 | 0.2-0.4 |
| Hepatitis B can be transmitted through sexual contact. | 37% (n=88) | 68% (n=221) | 0.3 | 0.000 | 0.2-0.4 |
| Hepatitis B can also be transmitted from mother to child. | 27% (n=63) | 56% (n=181) | 0.3 | 0.000 | 0.2-0.4 |
| Chronic Hepatitis can be treated successfully. | 26% (n=62) | 51% (n=162) | 0.3 | 0.000 | 0.2-0.5 |
| **Time in Germany** | **< 5 years in GER** | **≥ 5 years in GER** | **OR** | **p-value** | **95%-CI** |
| Hepatitis is a disease of the liver caused by a virus. | 50% (n=118) | 64% (n=207) | 0.6 | 0.002 | 0.4-0.8 |
| Hepatitis B and C can be transmitted through blood or used needles. | 47% (n=111) | 59% (n=189) | 0.6 | 0.007 | 0.4-0.9 |
| **Language skills ²** | **Language skills ↑** | **Language skills ↓** | **OR** | **p-value** | **95%-CI** |
| Hepatitis is a disease of the liver caused by a virus. | 66% (n=204) | 48% (n=123) | 2.1 | 0.000 | 1.5-2.9 |
| Hepatitis B and C can be transmitted through blood or used needles. | 62% (n=192) | 43% (n=109) | 2.2 | 0.000 | 1.6-3.1 |
| Hepatitis B can be transmitted through sexual contact. | 59% (n=182) | 50% (n=127) | 1.5 | 0.025 | 1.1-2.1 |
| Hepatitis B can also be transmitted from mother to child. | 50% (n=154) | 36% (n=91) | 1.8 | 0.001 | 1.3-2.5 |
| Chronic Hepatitis can be treated successfully. | 47% (n=142) | 32% (n=82) | 1.8 | 0.001 | 1.3-2.6 |
| **Health insurance in Germany** | **available** | **not available** | **OR** | **p-value** | **95%-CI** |
| Hepatitis is a disease of the liver caused by a virus. | 61% (n=271) | 40% (n=36) | 2.4 | 0.000 | 1.5-3.8 |
| Hepatitis B and C can be transmitted through blood or used needles. | 55% (n=243) | 40% (n=36) | 1.9 | 0.006 | 1.2-3.0 |
| Hepatitis B can be transmitted through sexual contact. | 57% (n=250) | 44% (n=40) | 1.7 | 0.019 | 1.1-2.7 |
| **Steady/permanent relationship** | **yes** | **no** | **OR** | **p-value** | **95%-CI** |
| Hepatitis B can also be transmitted from mother to child. | 47% (n=150) | 37% (n=79) | 1.5 | 0.022 | 1.1-2.2 |

* Mode of administration and age were not statistically significant

^1^ Lower education: Primary/ Secondary school/ No or other certificate. Higher education: High/ Vocational school/ University/ college.

^2^ Good skills: mother tongue, reported ‘very good’ or ‘good’ skills. Poor skills: reported ‘satisfactory’, ‘little’ or ‘none’ skills.

Table S2: Knowledge on general information about HIV (participants who responded „I knew this already“) by basic demographic information, partnership status and mode of administration. Significant differences only (p<0.05)*

| **Gender** | **Men** | **Women** | **OR** | **p-value** | **95%-CI** |
| --- | --- | --- | --- | --- | --- |
| You cannot tell from someone’s appearance whether he or she has HIV or not. | 81% (n=263) | 88% (n=214) | 0.6 | 0.022 | 0.4-0.9 |
| In Hamburg. you can get tested for HIV – anonymously and for free. | 42% (n=136) | 30% (n=71) | 1.7 | 0.002 | 1.2-2.5 |
| **Religion** | **Christianity** | **Islam** | **OR** | **p-value** | **95%-CI** |
| You cannot tell from someone’s appearance whether he or she has HIV or not. | 88% (n=287) | 75% (n=143) | 2.4 | 0.000 | 1.5-3.9 |
| There is a test which shows whether someone is HIV positive or not. | 97% (n=316) | 90% (n=172) | 3.9 | 0.001 | 1.7-8.8 |
| Africans are NOT deported from Germany just for having HIV. | 62% (n=196) | 46% (n=85) | 1.9 | 0.000 | 1.3-2.8 |
| **Education ^1^** | **Education ↓** | **Education ↑** | **OR** | **p-value** | **95%-CI** |
| You cannot tell from someone’s appearance whether he or she has HIV or not. | 79% (n=188) | 88% (n=287) | 0.5 | 0.003 | 0.3-0.8 |
| **Age** | **≤ 30 years old** | **> 30 years old** | **OR** | **p-value** | **95%-CI** |
| You cannot tell from someone’s appearance whether he or she has HIV or not. | 81% (n=222) | 87% (n=239) | 0.6 | 0.047 | 0.4-1.0 |
| Africans are NOT deported from Germany just for having HIV. | 46% (n=124) | 65% (n=175) | 0.5 | 0.000 | 0.3-0.7 |
| **Time in Germany** | **< 5 years in GER** | **≥ 5 years in GER** | **OR** | **p-value** | **95%-CI** |
| There is a test which shows whether someone is HIV positive or not. | 90% (n=213) | 99% (n=321) | 0.1 | 0.000 | 0.1-0.4 |
| Africans are NOT deported from Germany just for having HIV. | 46% (n=105) | 64% (n=206) | 0.5 | 0.000 | 0.3-0.7 |
| **Language skills ²** | **Language skills ↑** | **Language skills ↓** | **OR** | **p-value** | **95%-CI** |
| You cannot tell from someone’s appearance whether he or she has HIV or not. | 89% (n=275) | 78% (n=201) | 2.3 | 0.000 | 1.4-3.6 |
| Africans are NOT deported from Germany just for having HIV. | 66% (n=202) | 43% (n=108) | 2.6 | 0.000 | 1.8-3.6 |
| In Hamburg. you can get tested for HIV – anonymously and for free. | 43% (n=131) | 29% (n=75) | 1.8 | 0.001 | 1.3-2.6 |
| **Health insurance in Germany** | **available** | **not available** | **OR** | **p-value** | **95%-CI** |
| AIDS is caused by a virus called HIV. | 98% (n=434) | 89% (n=83) | 6.5 | 0.000 | 2.5-17.1 |
| You cannot tell from someone’s appearance whether he or she has HIV or not. | 87% (n=384) | 69% (n=64) | 2.9 | 0.000 | 1.8-4.9 |
| There is a test which shows whether someone is HIV positive or not. | 97% (n=428) | 85% (n=79) | 5.1 | 0.000 | 2.3-10.9 |
| Africans are NOT deported from Germany just for having HIV. | 57% (n=248) | 44% (n=40) | 1.7 | 0.016 | 1.1-2.7 |
| **Steady/permanent relationship** | **yes** | **no** | **OR** | **p-value** | **95%-CI** |
| You cannot tell from someone’s appearance whether he or she has HIV or not. | 87% (n=279) | 80% (n=176) | 1.6 | 0.050 | 1.0-2.5 |
| There is a test which shows whether someone is HIV positive or not. | 98% (n=315) | 90% (n=198) | 4.8 | 0.000 | 2.0-11.4 |
| In Hamburg, you can get tested for HIV – anonymously and for free. | 41% (n=131) | 30% (n=65) | 1.6 | 0.009 | 1.1-2.4 |

* Mode of questionnaire administration was not statistically significant

^1^ Lower education: Primary/ Secondary school/ No or other certificate. Higher education: High/ Vocational school/ University/ college.

^2^ Good skills: mother tongue, reported ‘very good’ or ‘good’ skills. Poor skills: reported ‘satisfactory’, ‘little’ or ‘none’ skills.

Table S3: Knowledge on specific information about HIV (participants who responded „I knew this already“) by basic demographic information, partnership status and mode of administration. Significant differences only (p<0.05)*

| **Gender** | **Men** | **Women** | **OR** | **p-value** | **95%-CI** |
| --- | --- | --- | --- | --- | --- |
| There is no cure for HIV infection. | 89% (n=281) | 96% (n=231) | 0.4 | 0.005 | 0.2-0.8 |
| **Religion** | **Christianity** | **Islam** | **OR** | **p-value** | **95%-CI** |
| There is no cure for HIV infection. | 95% (n=305) | 87% (n=160) | 2.8 | 0.001 | 1.5-5.3 |
| There are medications that can help people with HIV stay healthy. | 94% (n=300) | 85% (n=158) | 2.6 | 0.002 | 1.4-4.7 |
| HIV is not transmitted through kissing or shaking hands. | 93% (n=295) | 87% (n=158) | 2.0 | 0.020 | 1.1-3.7 |
| **Education ¹** | **Education ↓** | **Education ↑** | **OR** | **p-value** | **95%-CI** |
| There are medications that can help people with HIV stay healthy. | 87% (n=202) | 93% (n=299) | 0.5 | 0.022 | 0.3-0.9 |
| HIV is not transmitted through kissing or shaking hands. | 87% (n=199) | 94% (n=298) | 0.4 | 0.004 | 0.2-0.8 |
| **Age** | **≤ 30 years old** | **> 30 years old** | **OR** | **p-value** | **95%-CI** |
| There is no cure for HIV infection. | 89% (n=238) | 96% (n=260) | 0.4 | 0.005 | 0.2-0.8 |
| Time in Germany | < 5 years in GER | ≥ 5 years in GER | OR | p-value | 95%-CI |
| There is no cure for HIV infection. | 85% (n=195) | 97% (n=313) | 0.2 | 0.000 | 0.1-0.4 |
| **Language skills ^2^** | **Language skills ↑** | **Language skills ↓** | **OR** | **p-value** | **95%-CI** |
| There are medications that can help people with HIV stay healthy. | 94% (n=286) | 85% (n=216) | 2.9 | 0.000 | 1.6-5.3 |
| **Health insurance in Germany** | **available** | **not available** | **OR** | **p-value** | **95%-CI** |
| There is no cure for HIV infection. | 94% (n=415) | 81% (n=72) | 3.9 | 0.000 | 2.0-7.6 |
| There are medications that can help people with HIV stay healthy. | 94% (n=411) | 74% (n=66) | 5.1 | 0.000 | 2.8-9.4 |
| **Steady/permanent relationship** | **yes** | **no** | **OR** | **p-value** | **95%-CI** |
| There is no cure for HIV infection. | 96% (n=304) | 86% (n=185) | 3.5 | 0.000 | 1.8-6.8 |
| HIV is not transmitted through kissing or shaking hands. | 93% (n=293) | 86% (n=184) | 2.1 | 0.011 | 1.2-3.8 |

* Mode of questionnaire administration was not statistically significant

^1^ Lower education: Primary/ Secondary school/ No or other certificate. Higher education: High/ Vocational school/ University/ college.

^2^ Good skills: mother tongue, reported ‘very good’ or ‘good’ skills. Poor skills: reported ‘satisfactory’, ‘little’ or ‘none’ skills.

TableS4: Knowledge on STI “Which of the sexually transmitted diseases have you heard about before? by basic demographic information, partnership status and mode of administration. Significant differences only (p<0.05)*

| **Gender** | **Men** | **Women** | **OR** | **p-value** | **95%-CI** |
| --- | --- | --- | --- | --- | --- |
| Syphilis | 70% (n=198) | 83% (n=190) | 0.5 | 0.000 | 0.3-0.7 |
| Chlamydia | 20% (n=57) | 45% (n=102) | 0.3 | 0.000 | 0.2-0.5 |
| None known | 13% (n=41) | 6% (n=14) | 2.3 | 0.007 | 1.2-4.4 |
| **Religion** | **Christianity** | **Islam** | **OR** | **p-value** | **95%-CI** |
| Gonorrhoea | 72% (n=218) | 60% (n=94) | 1.7 | 0.010 | 1.1-2.5 |
| Syphilis | 78% (n=238) | 70% (n=109) | 1.6 | 0.037 | 1.0-2.5 |
| Chlamydia | 36% (n=108) | 15% (n=24) | 3.1 | 0.000 | 1.9-5.0 |
| All known | 7% (n=23) | 2% (n=4) | 3.6 | 0.014 | 1.2-10.5 |
| None known | 7% (n=21) | 18% (n=34) | 0.3 | 0.000 | 0.2-0.6 |
| **Education ^1^** | **Education ↓** | **Education ↑** | **OR** | **p-value** | **95%-CI** |
| Syphilis | 68% (n=134) | 81% (n=253) | 0.5 | 0.001 | 0.3-0.8 |
| Herpes | 27% (n=53) | 42% (n=130) | 0.5 | 0.001 | 0.4-0.8 |
| Genital Warts | 24% (n=48) | 35% (n=110) | 0.6 | 0.010 | 0.4-0.9 |
| Chlamydia | 20% (n=40) | 38% (n=118) | 0.4 | 0.000 | 0.3-0.6 |
| All known | 2% (n=4) | 10% (n=31) | 0.2 | 0.000 | 0.1-0.5 |
| None known | 18% (n=42) | 4% (n=13) | 5.1 | 0.000 | 2.7-9.8 |
| **Age** | **≤ 30 years old** | **> 30 years old** | **OR** | **p-value** | **95%-CI** |
| Gonorrhoea | 64% (n=156) | 76% (n=191) | 0.6 | 0.004 | 0.4-0.8 |
| Syphilis | 70% (n=172) | 81% (n=206) | 0.5 | 0.003 | 0.4-0.8 |
| Chlamydia | 36% (n=89) | 26% (n=66) | 1.6 | 0.014 | 1.1-2.4 |
| **Time in Germany** | **< 5 years in GER** | **≥ 5 years in GER** | **OR** | **p-value** | **95%-CI** |
| Gonorrhoea | 59% (n=123) | 77% (n=232) | 0.4 | 0.000 | 0.3-0.7 |
| Syphilis | 68% (n=141) | 81% (n=246) | 0.5 | 0.001 | 0.3-0.7 |
| Herpes | 29% (n=61) | 41% (n=123) | 0.6 | 0.009 | 0.4-0.9 |
| Chlamydia | 26% (n=54) | 35% (n=105) | 0.7 | 0.037 | 0.4-0.977 |
| None known | 13% (n=31) | 7% (n=22) | 2.1 | 0.013 | 1.2-3.6 |
| **Language skills ²** | **Language skills ↑** | **Language skills ↓** | **OR** | **p-value** | **95%-CI** |
| Herpes | 43% (n=123) | 27% (n=61) | 2.0 | 0.000 | 1.4-2.9 |
| Genital Warts | 34% (n=99) | 26% (n=58) | 1.5 | 0.039 | 1.0-2.2 |
| None known | 7% (n=21) | 13% (n=34) | 0.5 | 0.011 | 0.3-0.9 |
| **Health insurance in Germany** | **yes** | **No/nor sure** | **OR** | **p-value** | **95%-CI** |
| Syphilis | 82% (n=338) | 47% (n=34) | 5.3 | 0.000 | 3.1-9.0 |
| Herpes | 38% (n=157) | 25% (n=18) | 1.9 | 0.026 | 1.1-3.3 |
| Chlamydia | 35% (n=143) | 16% (n=12) | 2.7 | 0.002 | 1.4-5.2 |
| None known | 7% (n=31) | 22% (n=21) | 0.3 | 0.000 | 0.1-0.5 |
| **Steady/permanent relationship** | **yes** | **no** | **OR** | **p-value** | **95%-CI** |
| Chlamydia | 36% (n=102) | 27% (n=54) | 1.5 | 0.048 | 1.0-2.2 |
| **Mode of Administration** | **Interview** | **Self-completion** | **OR** | **p-value** | **95%-CI** |
| Gonorrhoea | 83% (n=206) | 57% (n=150) | 3.6 | 0.000 | 2.4-5.5 |
| Genital Warts | 26% (n=64) | 36% (n=94) | 0.6 | 0.015 | 0.4-0.9 |
| Chlamydia | 39% (n=96) | 24% (n=63) | 2.0 | 0.000 | 1.4-2.9 |
| None known | 14% (n=39) | 6% (n=16) | 2.6 | 0.002 | 1.4-4.7 |

^1^ Lower education: Primary/ Secondary school/ No or other certificate. Higher education: High/ Vocational school/ University/ college.

^2^ Good skills: mother tongue, reported ‘very good’ or ‘good’ skills. Poor skills: reported ‘satisfactory’, ‘little’ or ‘none’ skills.
