# Supplementary material for: Determinants of HIV, viral hepatitis and STI prevention needs among African migrants in Germany; a cross-sectional survey on knowledge, attitudes, behaviors and practices
Source: BMC Public Health. 2015 Aug 6;15:753. doi: 10.1186/s12889-015-2098-2 (PMC4545823; doi:10.1186/s12889-015-2098-2)
Supplement: Additional file 2: — Results of stratified analysis of sexual behavior by school education, age group and religion, HIV/STI-testing history by school education, age and religion and attitudes toward PLWH by age and religion. (DOCX 31 kb) [file 12889_2015_2098_MOESM2_ESM.docx]

**Additional file 2**

Results of stratified analysis of sexual behavior by school education, age group and religion, HIV/STI-testing history by school education, age and religion and attitudes toward PLWH by age and religion

Table S1: Results of stratified analysis of sexual behavior by school education, age and religion

| School education | **Education ↓** | **Education ↑** | **OR** | **p-value** | **95%-CI** |
| --- | --- | --- | --- | --- | --- |
| Ever had sex | 96.6% (n=310) | 94.8% (n=217) | 0.6 | 0.295 | 0.3-1.5 |
| Sex within the last 12 months | 90.3% (n=280) | 88.5% (n=192) | 0.8 | 0.496 | 0.5-1.4 |
| Single sex partner (12 months) | 62.5% (n=173) | 56.1% (n=106) | 0.8 | 0.168 | 0.5-1-1 |
| More than 5 sex partners (12 months) | 2.2% (n=6) | 4.2% (n=8) | 2.0 | 0.199 | 0.7-5.9 |
| Steady/ permanent sexual partner(s) | 78.1% (n=214) | 66.3% (n=124) | 0.6 | 0.005 | 0.4-0.8 |
| Steady/ permanent sexual partner(s) from Germany | 31.1% (n=66) | 39.8% (n=49) | 1.5 | 0.106 | 0.9-2.3 |
| Sex with casual partner(s) | 38.1% (n=61) | 30.0% (n=74) | 1.4 | 0.087 | 0.9-2.2 |
| Inconsistent condom use with casual partners | 61.1% (n=44) | 68.3% (n=41) | 1.4 | 0.388 | 0.7-2-8 |
| Sexual violence (once and/or repeated) | 9.4% (n=30) | 10.7% (n=24) | 1.2 | 0.624 | 0.7-2-0 |
| Age | **≤ 30 years old** | **> 30 years old** | **OR** | **p-value** | **95%-CI** |
| Ever had sex | 92.9% (n=247) | 99.6% (n=266) | 0.1 | 0.000 | 0.0-0.4 |
| Sex within the last 12 months | 87.9% (n=217) | 91.7% (n=244) | 0.7 | 0.146 | 0.4-1.2 |
| Single sex partner (12 months) | 49.8% (n=107) | 67.9% (n=163) | 0.5 | 0.000 | 0.3-0.7 |
| More than 5 sex partners (12 months) | 3.3% (n=7) | 2.9% (n=7) | 1.1 | 0.834 | 0.4-3.3 |
| Steady/ permanent sexual partner(s) | 64.5% (n=136) | 80.8% (n=194) | 0.4 | 0.000 | 0.3-0.7 |
| Steady/ permanent sexual partner(s) from Germany | 43.0% (n=58) | 28.6% (n=55) | 1.9 | 0.007 | 1.2-3.0 |
| Sex with casual partner(s) | 39.8% (n=68) | 29.1% (n=66) | 1.6 | 0.025 | 1.1-2.5 |
| Inconsistent condom use with casual partners | 66.7% (n=44) | 63.1% (n=41) | 1.2 | 0.667 | 0.6-2.4 |
| Sexual violence (once and/or repeated) | 9.5% (n=25) | 10.6% (n=28) | 0.9 | 0.664 | 0.5-1.6 |
| Religion | **Christianity** | **Islam** | **OR** | **p-value** | **95%-CI** |
| Ever had sex | 97.4% (n=303) | 91.7% (n=166) | 3.4 | 0.004 | 1.4-8.2 |
| Sex within the last 12 months | 90.1% (n=273) | 88.6% (n=147) | 1.2 | 0.601 | 0.6-2.2 |
| Single sex partner (12 months) | 64.9% (n=174) | 55.2% (n=80) | 1.5 | 0.052 | 0.9-2.3 |
| More than 5 sex partners (12 months) | 3.4% (n=9) | 3.4% (n=5) | 1.0 | 0.961 | 0.3-3.0 |
| Steady/ permanent sexual partner(s) | 77.9% (n=208) | 67.8% (n=97) | 1.7 | 0.026 | 1.1-2.6 |
| Steady/ permanent sexual partner(s) from Germany | 29.6% (n=61) | 43.8% (n=42) | 0.5 | 0.016 | 0.3-0.9 |
| Sex with casual partner(s) | 29.0% (n=69) | 38.9% (n=49) | 0.6 | 0.055 | 0.4-1.1 |
| Inconsistent condom use with casual partners | 73.5% (n=50) | 63.8% (n=30) | 1.6 | 0.266 | 0.7-3.5 |
| Sexual violence (once and/or repeated) | 12.4% (n=38) | 6.7% (n=12) | 2.0 | 0.046 | 1.1-3.9 |

Table S2: Results of stratified analysis of HIV/STI-testing history school education, age and religion

| School education | Education ↓ | Education ↑ | OR | p-value | | 95%-CI | |
| --- | --- | --- | --- | --- | --- | --- | --- |
| Ever tested for HIV | 68.9% (n=226) | 65.0% (n=152) | 0.8 | | 0.326 | | 0.6-1.2 |
| Ever tested for an STI | 42.3% (n=126) | 29.3% (n=54) | 1.8 | | 0.004 | | 1.2-2.6 |
| Ever diagnosed with an STI | 48.1% (n=52) | 50.0% (n=19) | 0.9 | | 0.844 | | 0.4-1.9 |
| Age | **≤ 30 years old** | **> 30 years old** | **OR** | **p-value** | | **95%-CI** | |
| Ever tested for HIV | 61.3% (n=168) | 72.7% (n=197) | 0.6 | | 0.005 | | 0.4-0.9 |
| Ever tested for an STI | 33.6% (n=78) | 41.0% (n=98) | 1.4 | | 0.098 | | 0.9-2.0 |
| Ever diagnosed with an STI | 41.9% (n=26) | 53.7% (n=44) | 1.6 | | 0.163 | | 0.8-3.1 |
| Religion | **Christianity** | **Islam** | **OR** | **p-value** | | **95%-CI** | |
| Ever tested for HIV | 71.5% (n=226) | 62.0% (n=116) | 1.5 | | 0.028 | | 1.1-2.3 |
| Ever tested for an STI | 41.8% (n=119) | 26.8% (n=38) | 0.5 | | 0.002 | | 0.3-0.8 |
| Ever diagnosed with an STI | 49.0% (n=48) | 46.9% (n=15) | 0.9 | | 0.836 | | 0.4-2.0 |

Table S3: Results of stratified analysis of attitudes toward PLWH by age and religion

| Age | ≤ 30 years old | > 30 years old | OR | p-value | 95%-CI |
| --- | --- | --- | --- | --- | --- |
| I treat them like any other person. | 70.1% (n=169) | 80.7% (n=209) | 0.6 | 0.006 | 0.4-0.9 |
| I avoid physical contact. | 13.3% (n=32) | 10.4% (n=27) | 1.3 | 0.323 | 0.8-2.3 |
| I avoid being seen with this person. | 8.7% (n=21) | 3.5% (n=9) | 2.7 | 0.014 | 1.2-5.9 |
| I blame this person secretly. | 8.7% (n=21) | 11.6% (n=30) | 0.7 | 0.289 | 0.4-1.3 |
| I behave differently. | 4.6% (n=11) | 5.0% (n=13) | 0.9 | 0.812 | 0.4-2.1 |
| Religion | **Christianity** | **Islam** | **OR** | **p-value** | **95%-CI** |
| I treat them like any other person. | 76.9% (n=223) | 71.4% (n=120) | 1.3 | 0.193 | 0.9-2.1 |
| I avoid physical contact. | 10.7% (n=31) | 14.3% (n=24) | 0.7 | 0.254 | 0.4-1.3 |
| I avoid being seen with this person. | 5.2% (n=15) | 7.7% (n=13) | 0.7 | 0.269 | 0.3-1.4 |
| I blame this person secretly. | 10.7% (n=31) | 10.1% (n=17) | 1.1 | 0.848 | 0.6-2.0 |
| I behave differently. | 4.8% (n=14) | 4.8% (n=8) | 1.0 | 0.975 | 0.4-2.5 |
